# The Use of Electronic Cigarettes in Saudi Arabia: A Narrative Review

**DOI:** 10.7759/cureus.54167

**Published:** 2024-02-14

**Authors:** Abdullah Alhalafi

**Affiliations:** 1 Department of Family and Community Medicine, College of Medicine, University of Bisha, Bisha, SAU

**Keywords:** tobacco-related diseases, literature review, smoking trends, children and young adults, prevalence, public health, saudi arabia, tobacco consumption, e-cigarette use, electronic cigarettes

## Abstract

The use of electronic cigarettes (or "e-cigarettes") is spreading throughout the world. Population-level data from a number of countries indicate that e-cigarettes are used more by children and young adults than by the overall population. Although extensive research has been conducted in Western nations to better understand many aspects of e-cigarette usage among children and young adults, Middle Eastern nations have little data on this topic.

The use of tobacco is detrimental to the health, finances, and national spirit of Saudi Arabia. More than 7,000 of its citizens die each year from diseases caused by tobacco use. Nevertheless, more than 20,000 youngsters and 3,352,000 adults smoke cigarettes each day. Similarly, the use of electronic cigarettes is on the rise, possibly as a result of aggressive publicity and marketing campaigns by manufacturers that appeal to the younger demographic. This review was undertaken through literature research to ascertain the usage and attitudes toward e-cigarette use among the Saudi population.

## Introduction and background

Tobacco usage is widespread globally. Manufacturers of these products demonstrate exceptional marketing prowess, employing strong strategies to promote their products through appealing advertisements and product displays. The FDA defines electronic cigarettes, sometimes referred to as e-cigarettes, as battery-operated devices capable of delivering nicotine and other substances [1]. E-cigarettes are commonly misperceived as being less toxic than tobacco products, despite being recognized as hazardous and deleterious to the respiratory system [2]. However, they are widely used and promoted worldwide, particularly among young adults, including those from wealthy homes [3]. Unfortunately, media influences contribute to the increasing prevalence of e-smoking by claiming that it is relatively less harmful than other kinds of smoking [4]. Individuals of both genders across various age groups, including the young, adults, and seniors, are showing a growing interest in these products, with youngsters being the primary target. In addition, young individuals are attracted to e-cigarettes due to the belief that they can assist in quitting tobacco smoking [5]. This phenomenon could be attributed to the appeal and influence of developing trends on young adults, especially if they believe that these trends can help them overcome the hazardous habit of tobacco smoking. Moreover, the wide range of flavors makes them quite popular among most users [6]. Furthermore, individuals who are incapable of quitting smoking believe that e-cigarettes offer a better option with reduced health hazards [7,8]. Another notable danger associated with e-cigarettes is their potential to cause cancer [7]. Recent research has shown that chemical analysis of e-cigarettes has detected several carcinogens, substantially elevating the risk of cancer development [8]. This could potentially enhance vulnerability to respiratory conditions such as asthma and chronic obstructive pulmonary disease, in addition to the hazards linked to exposure to secondhand smoke [9]. Despite the abundance of data on the risks associated with tobacco smoking and e-cigarettes, there is a dearth of knowledge concerning the extent of e-cigarette usage in Saudi Arabia and the factors contributing to its increasing popularity in the country [10]. Therefore, this review was conducted to assess the prevalence and perception of e-cigarette use among the Saudi population by analyzing existing literature.

## Review

History of e-cigarettes and worldwide assumptions

Electronic cigarettes and vapes were first introduced in the United States in 2007 [11]. Since then, their sales have grown significantly, leading the sector to reach a worth of over two billion USD in 2013. The increase in the use of e-cigarettes has occurred simultaneously with an upsurge in smoking cessation rates and a decrease in the prevalence of smoking. According to a 2015 survey, more than 20% of American individuals between the ages of 18 and 24 had experimented with e-cigarettes [11]. In 2014, e-cigarettes were used by 47.6% of active cigarette smokers and 55.4% of individuals who had recently quit smoking in the United States [12]. This data suggests that over half of both groups had experimented with e-cigarettes. As a result, electronic cigarettes and vapes have been significant focuses of research to determine their effectiveness in helping people quit smoking, as well as to examine the comparative effects of electronic cigarettes and vapes versus traditional cigarette smoking. Currently, comprehensive investigations have been conducted on several facets of electronic cigarettes. The public has engaged in discussions on the prevalence of e-cigarette consumption, attitudes towards e-cigarettes, assessments of awareness, and factual information concerning e-cigarettes [13]. Furthermore, scientists have conducted comprehensive analyses and evaluations of e-cigarettes, including scrutinizing the levels of inflammation biomarkers present in e-cigarettes, examining the usage of e-cigarettes among individuals with mental health conditions, and investigating the presence of the flavoring chemical diacetyl, which is associated with bronchiolitis obliterans [14]. Although there is a wealth of research on e-cigarettes that generally indicates harmful consequences, several researchers advocate for a different viewpoint. They argue that vaping is relatively 'less detrimental' than smoking, which has been conclusively associated with different ailments such as lung cancer, COPD, and interstitial lung disease [14]. Scientists are currently undertaking meticulous examinations of e-cigarettes in response to increasing concerns about the potential normalization of tobacco product consumption [15]. As a result of the significant increase in e-cigarette usage, multiple studies have been conducted to evaluate different aspects related to the composition, functioning, and negative consequences of e-cigarette consumption [15]. However, some phenomena have not yet been proven through empirical evidence. A pressing concern faced by researchers is the approval of e-cigarettes for commercial distribution without adequate assessment, considering the novelty of these devices. Moreover, the lack of factual proof supporting the negative effects of using e-cigarettes for an extended period is a key reason for their popularity, as they have been widely advertised as a safer alternative to regular smoking. However, additional investigation is required in this field to determine whether vaping genuinely facilitates smoking cessation. A significant majority of individuals, particularly young individuals, are presently using electronic cigarettes without any knowledge of the composition of these devices or the precise substances they contain [13].

Impact of e-cigarettes in Saudi Arabia: composition and economic effect

The excessive use of tobacco adversely affects health and hinders the socio-economic development of Saudi Arabia. Over 7,000 individuals succumb to diseases attributed to tobacco use [16]. Furthermore, approximately 20,000 individuals in the age group of 10 to 14, together with 3,352,000 individuals aged 15 and above, persist in engaging in tobacco consumption [16]. The estimated economic impact of tobacco smoking in Saudi Arabia is predicted to be roughly 4,545 million riyals [17]. This includes explicit costs directly related to healthcare expenses, and implicit costs resulting from reduced productivity caused by premature death and illness. Multiple e-cigarette designs are currently available in the market. However, the fundamental components of an e-cigarette consist of a battery, a compartment containing e-juice (a liquid that includes flavorants, a solvent, and nicotine), a heating element, and a mouthpiece [18]. The battery regulates the heating element, which warms the e-juice and produces an aerosol breathed through the mouthpiece. A study has revealed that e-liquids utilized in e-cigarettes predominantly consist of flavored variants, with around 7,700 distinct flavors identified [19]. These flavors are primarily characterized by fruit or candy profiles. The extensive accessibility and widespread appeal of flavored e-cigarettes raise significant concerns over their potential impact on public health. The main issue for young people is the accessibility of e-cigarettes that come in sugary flavors, making it easier for them to become addicted to nicotine and imitate smoking. This increases the likelihood of them transitioning to traditional tobacco products. Manufacturers have been employing these flavors for many years to entice young people to use tobacco products, typically serving to conceal the taste and harshness of these products.

Impact of e-cigarettes on health

The increasing prevalence of e-cigarettes has emerged as a notable public health issue. This is because e-cigarettes release aerosols containing compounds that can potentially be toxic. The predominant components in e-cigarette aerosols are propylene glycol, glycerin, hazardous metals like lead, cadmium, nickel, and carcinogenic carbonyl compounds, including formaldehyde [20].

These substances can potentially harm DNA, decreasing its capacity for self-repair during replication [20]. Additionally, they can induce respiratory illness. Moreover, nicotine has been demonstrated to be detrimental, particularly in young individuals who are still developing and in pregnant women [21]. The effects of prolonged exposure to nicotine may be more noticeable in adolescent users, leading to difficulties in paying attention, mood disturbances, and overall cognitive impairment [21]. Remarkably, nicotine has demonstrated a consistent ability to decrease insulin sensitivity across all age groups, potentially leading to the development of insulin resistance and type II diabetes [22]. A notable characteristic of the latest generation of devices is their inclusion of larger batteries and enhanced capacity to heat the liquid to higher temperatures. This has the potential to increase the release of nicotine, generate additional toxic substances, and result in exceedingly elevated levels of formaldehyde, a well-established carcinogenic compound. The degree of tolerance exhibited by consumers towards the flavor of the aerosol when heated to this specific temperature is a subject of ongoing dispute [23].

Given the knowledge of the health implications of e-cigarettes, addressing this issue in Saudi Arabia is necessary. The harmful effects of e-cigarettes have been overlooked in the ongoing debate, despite their widespread use and the targeting of young people by manufacturers' marketing strategies. Therefore, this review evaluates the frequency and public opinion of e-cigarette usage among the Saudi population by analyzing the available literature.

Beliefs, perceptions, attitudes, and awareness about e-cigarettes in Saudi Arabia

Prevalence of E-cigarettes Among the Saudi population

Although Saudi Arabia is making significant efforts to reduce tobacco smoking, researchers have acknowledged that the frequency of tobacco smoking is concerning and requires prompt action from both Saudi officials and health professionals. Two national surveys conducted in 2013 and 2018 revealed that the prevalence rate of tobacco smoking among the Saudi population was 12.2% and 21.4%, respectively [24]. The 9.2% increase in tobacco prevalence over just five years suggests a deficiency in addressing the current tobacco problem. Saudi college students exhibit a higher prevalence of tobacco consumption compared to the overall Saudi population [24]. A systematic review and meta-analysis conducted from 2010 to 2018 revealed that the smoking prevalence among college students in Saudi Arabia was 17% [25]. The meta-analysis showed a 21% disparity between the smoking rates of Saudi males and females, with the former exhibiting a higher prevalence [25]. Another review emphasized the significance of closely monitoring tobacco smoking among young people to prevent it from escalating to a point where it becomes a financial and healthcare burden. Alotaibi SA et al. presented an epidemiological framework about the incidence of tobacco smoking among college students in the Kingdom of Saudi Arabia (KSA) [25]. Almutham A et al. did a study on 229 medical students in Buraydah, Saudi Arabia and observed that 10.6% of the participants used e-cigarettes [26]. Qanash S et al. conducted a separate investigation involving students in Jeddah's health science colleges [27]. The survey form received responses from a total of 1,007 students. The researchers documented a prevalence rate of 27.7% for e-cigarette usage among individuals [27]. Alzalabani AA and Eltaher SM conducted an independent investigation on 527 medical students in Medina [28]. The researchers recorded a prevalence rate of 15.9% for the use of e-cigarettes among the participants in the study. The results also showed a greater occurrence among male participants than female participants, with statistical significance (p < 0.05) [28]. Aqeeli AA et al. conducted a study with 775 students from Jazan University. Their research discovered that 21.0% of the study participants used e-cigarettes [29]. Habib E et al. conducted a study including 401 medical students in Riyadh [30]. The researchers recorded a prevalence rate of 12.2% for the use of e-cigarettes among the participants in the study [30]. The findings also suggested that the prevalence was three times higher among males than females [30].

Factors of E-cigarette Consumption

The surge in e-cigarette popularity can be attributed to multiple factors stemming from its widespread appeal across different age demographics. Furthermore, the combination of e-cigarette advertising and the limited amount of thorough investigation has influenced the perception of e-cigarettes as a viable substitute for traditional smoking cessation methods.

The Alzahrani study reveals that e-cigarettes have gained significant popularity among young adults due to their multifaceted success [31]. The primary motivation for using e-cigarettes was amusement, accounting for 33.9% of users. Similarly, traditional smoking had a 37.1% user rate for entertainment purposes. On the other hand, sorrow and despair were identified as the least prevalent factors, accounting for only 4.8% among electronic cigarette users and 10.3% among conventional smoking users [31]. A survey conducted by Qanash S et al. in the Jeddah region, focusing on medical students, revealed that a greater proportion of e-cigarette users (49%) cited entertainment as the primary motive for their e-cigarette consumption compared to the general public [27]. In addition, a greater proportion (7.8%) of medical students who suffer from depression were found to use electronic cigarettes, while 16.2% used conventional smoking for the same purpose [27]. Furthermore, the second most frequently cited motive for using e-cigarettes was to assist in quitting traditional smoking, but the prevalence of conventional smoking was attributed to the influence of the surrounding environment [27].

Research findings indicate that college students who associate with peers who currently engage in smoking are more prone to adopting smoking habits themselves [25]. Among Saudi college students, the smoking behavior of peers was found to be a significant predictor of smoking behavior in five out of the seven investigations [25]. Moreover, the presence of any individual who engages in smoking inside a household was found to be associated with the smoking behaviors of college students.

Amin HS et al. found that Saudi college students who were exposed to a high number of non-smoking media messages were less likely to smoke [32]. This exposure acted as a protective factor against smoking. Meanwhile, Jiang N et al. suggested that governmental measures on tobacco control indicated the transition from smoking to non-smoking behavior [33].

The higher prevalence of e-cigarette usage among medical students in Saudi Arabia can be attributed to the current surge and availability of e-cigarettes, combined with the users' belief that they carry fewer health hazards.

The Attitude of the Saudi Population Towards E-Cigarettes

The primary motivations for using e-cigarettes, as identified among the Saudi population, include the desire to decrease or quit traditional cigarette smoking, financial considerations, recreational purposes, and curiosity. A study indicates that Saudi individuals who use electronic cigarettes had a comparable level of knowledge (93.6%) about the potential repercussions [34]. This heightened awareness among Saudi vapers can be ascribed to escalated online marketing of vaping, along with the extensive circulation of promotional vaping videos on the internet. A study by Abu Khalid AS et al. revealed that around 20% of the participants believed that vaping does not present any hazards to overall and dental well-being [35]. Moreover, an overwhelming majority of 72.6% considered vaping to be a safer option compared to smoking conventional cigarettes. Additionally, their survey revealed that 88.6% of the participants perceived vaping as a highly effective method to quit smoking, and 71.7% of vapers confirmed that they had completely abandoned all other smoking alternatives after transitioning to electronic vaping [35].

Furthermore, Qanash S et al. observed that a significant proportion of medical students, specifically 35.9%, expressed strong agreement on the superiority of e-cigarettes over conventional smoking and tobacco products concerning patient health [27]. Moreover, this study revealed a divergence in viewpoints among the general community, with the majority of e-cigarette users expressing a wish to discontinue their e-cigarette consumption.

Meanwhile, in a research conducted by Abu Khalid AS et al., a staggering 79.2% of the general public expressed a strong desire to discontinue the use of e-cigarettes, indicating a remarkably high level of dissatisfaction [35]. In Saudi Arabia, 66.2% of medical students had contemplated discontinuing their use of e-cigarettes [35].

## Conclusions

In summary, the electronic cigarette sector in Saudi Arabia is thriving, providing a wide range of products to consumers. The increasing awareness of the harmful effects of traditional tobacco smoking, coupled with strict regulations on smoking in public spaces, has led to a rise in the popularity of e-cigarettes. While the sale of e-cigarettes containing nicotine is now banned, the industry for nicotine-free e-cigarettes and e-liquids is flourishing. With the growing prevalence of e-cigarettes, regulators and researchers must continuously monitor the industry and ensure the safety of consumers.

This review suggests that e-cigarettes may not have the degree of safety their consumers perceive. There is abundant information indicating that e-cigarettes are hazardous and present a comparable level of danger to traditional tobacco products. This situation is problematic and necessitates meticulous consideration. To assess the safety of e-cigarettes and their effectiveness in reducing the use of traditional cigarettes, additional research is required, specifically through well-designed clinical trials.
